# Hierarchical Partitioning of Metazoan Protein Conservation Profiles Provides New Functional Insights

**DOI:** 10.1371/journal.pone.0090282

**Published:** 2014-03-04

**Authors:** Jonathan Witztum, Erez Persi, David Horn, Metsada Pasmanik-Chor, Benny Chor

**Affiliations:** 1 Faculty of Life Sciences, Tel-Aviv University, Tel-Aviv, Israel; 2 School of Physics and Astronomy, Tel-Aviv University, Tel-Aviv, Israel; 3 School of Computer Science, Tel-Aviv University, Tel-Aviv, Israel; University of Lausanne, Switzerland

## Abstract

The availability of many complete, annotated proteomes enables the systematic study of the relationships between protein conservation and functionality. We explore this question based solely on the presence or absence of protein homologues (a.k.a. conservation profiles). We study 18 metazoans, from two distinct points of view: the human's and the fly's. Using the GOrilla gene ontology (GO) analysis tool, we explore functional enrichment of the “universal proteins”, those with homologues in all 17 other species, and of the “non-universal proteins”. A large number of GO terms are strongly enriched in both human and fly universal proteins. Most of these functions are known to be essential. A smaller number of GO terms, exhibiting markedly different properties, are enriched in both human and fly non-universal proteins. We further explore the non-universal proteins, whose conservation profiles are consistent with the “tree of life” (TOL consistent), as well as the TOL inconsistent proteins. Finally, we applied Quantum Clustering to the conservation profiles of the TOL consistent proteins. Each cluster is strongly associated with one or a small number of specific monophyletic clades in the tree of life. The proteins in many of these clusters exhibit strong functional enrichment associated with the “life style” of the related clades. Most previous approaches for studying function and conservation are “bottom up”, studying protein families one by one, and separately assessing the conservation of each. By way of contrast, our approach is “top down”. We globally partition the set of all proteins hierarchically, as described above, and then identify protein families enriched within different subdivisions. While supporting previous findings, our approach also provides a tool for discovering novel relations between protein conservation profiles, functionality, and evolutionary history as represented by the tree of life.

## Introduction

The relationship between sequence conservation and evolutionary functional constraints has been extensively studied in biology, starting as early as Eck and Dayhoff [1], and Kimura [2] and is still of great interest today [3], [4]. Following vast improvements in sequencing technologies, genomes of hundreds of species, including tens multi-cellular eukaryotes, were sequenced [5]. Consequently, complete collections of protein sequences of many species were derived (at different levels of reliability, see Lindblad-Toh et al. [6]), enabling comprehensive studies of proteomes, and a rigorous analysis of proteins' conservation.

The Gene Ontology (GO) project [7] has created a standard “vocabulary” for representing proteins' functions across a variety of species. Tools for computing GO enrichments were developed, and are routinely used to determine which GO terms (ontologies) are enriched, or over represented, in different protein subsets. These tools employ data that is periodically being updated, using new predictions or laboratory based evidence.

We explore the relations of metazoan conservation profiles and protein families' functional enrichment. To address this question, we should first determine which conservation measure is being used. The simplest conservation measure is binary: the presence or absence of homologues to a given protein sequence, across a set of species. This measure results in a zero-one vector per protein, which is known as the conservation profile of the protein. Conservation profiles were introduced and used mostly for inferring evolutionary histories [8]–[10]. Conservation profiles were also used for inferring and predicting organism-to-phenotype associations [11]. There are many conservation studies based on whole proteomes data [12], [13], but only a few works in this context explore a wide evolutionary range of species. These include the work of Martin et al. [14], where 58 bacterial proteomes are studied, and the work of Lopez-Bigas et al. [4], where 16 eukaryote proteomes are studied.

We study 18 metazoan species, whose evolutionary range is very similar to the one in Lopez-Bigas et al. [4]. While the methodology employed by Lopez-Bigas et al. [4] can be described as “bottom up”, ours can be described as “top down”. It provides a global view of protein conservation, in addition to studying protein families enriched within the different organism subdivisions.

We use the conservation profiles to hierarchically partition the set of proteins, and then apply a gene ontology analysis tool, GOrilla (Eden et al. [15]). This enables the elucidation of relations between sequence conservation, function, and the consistency of conservation profiles with the tree of life (TOL). Furthermore, we explore conservation from the points of view of two reference species, thus expanding the single point of view as in Martin et al. [14] or Lopez-Bigas et al. [4]. One reference species we use is human, the second one is Drosophila melanogaster. We demonstrate a rich structure of functional enrichment based on the conservation profiles, features shared by both viewpoints, as well as those where the two viewpoints give rise to differences. We remark that both human and Drosophila melanogaster have very closely related species in our study.

An additional novel aspect of our analysis is to study the “tree of life” consistent proteins, having homologues in a subset of the species that forms a clade in the tree of life. This allows the elucidation and discovery of relations between the “life style” of several clades, and functional enrichment of proteins whose conservation profiles are related to these clades. Some expected as well as unexpected (or unknown) enrichments are revealed using this approach.

## Results

We studied the conservation profile of each RefSeq protein in the two reference species, human and D. melanogaster, each with respect to 17 other metazoan species. We associated each protein with a conservation class, based on the number of species where it has homologous proteins (varying between 17 and 0), and examined whether the conservation classes are related to essential proteins. GO enrichment analysis tools were then applied to the universal protein class (those proteins having homologues in all 17 other metazoan species) from both human and fly points of view, and to the complementary set, the non-universal proteins of both species. We looked for similarities and differences of enriched functional terms from both viewpoints. Next, we examined the non-universal TOL consistent proteins, whose conservation profiles are consistent with the tree of life, as well as TOL inconsistent proteins. Finally, we applied quantum clustering [16] to the conservation profiles of the non-universal, TOL consistent proteins, and investigated GO enrichment in each resulting cluster.

### Conservation Classes and Essential Genes

In our setting, the number of species where a given human or fly RefSeq protein has homologous proteins is in the range 17 to 0 (denoted by *i*). For each reference species and each *i* in this range, the conservation class H_i_ is defined as the collection of proteins with homologous proteins in *i* species. With respect to each reference species, i.e., either human or fly (Table 1), universal proteins are defined as those in class H_17_, having homologous proteins in all other 17 species. The rest are non-universal proteins. In particular, orphan proteins are defined as those in class H_0_ (having no homologous proteins in any other species). Table 1 shows the distribution of proteins in all H_i_ classes, for both reference species. There are a total of 34,817 human RefSeq proteins, out of which 22,841 are universal and 81 are orphans. There are 22,309 fly RefSeq proteins, out of which 14,444 are universal, and 62 are orphans. In both cases, the frequency of universal proteins is approximately 65%, whereas the frequency of orphan proteins is approximately 0.25%. (We remark that the mapping from universal proteins in one species to a different species is not one to one.)

**Table 1:** Distribution of Human and Fly Proteins in the Classes H_i_.

H_i_	No. of Human Proteins	Percentage of Human Proteins	No. of Fly Proteins	Percentage of Fly proteins
**17**	22841	65.60%	14444	64.75%
**16**	2555	7.34%	1714	7.68%
**15**	1575	4.52%	755	3.38%
**14**	1296	3.72%	555	2.49%
**13**	1136	3.26%	329	1.47%
**12**	1068	3.07%	280	1.26%
**11**	1021	2.93%	237	1.06%
**10**	753	2.16%	266	1.19%
**9**	455	1.31%	251	1.13%
**8**	325	0.93%	331	1.48%
**7**	346	0.99%	369	1.65%
**6**	301	0.86%	415	1.86%
**5**	268	0.77%	522	2.34%
**4**	253	0.73%	567	2.54%
**3**	231	0.66%	526	2.36%
**2**	198	0.57%	417	1.87%
**1**	114	0.33%	269	1.21%
**0**	81	0.23%	62	0.28%

A gene or protein is called *essential* if the loss of its function reduces the fitness of the organism [17]. Essential genes are determined by a combination of clinical and experimental approaches. They were studied over a wide evolutionary range of both prokaryotes and eukaryotes, including human and fly. For fly, 1228 essential genes are known, while for human the number is 312. The substantial difference is due to the inapplicability of experimental methods, like knock out techniques, to humans.

We examined the distribution of human's and fly's essential genes with respect to the different conservation classes. Table 2 describes the number of essential genes per conservation class. We see that for both human and fly, the vast majority of essential proteins are universal. Most non-universal essential proteins are in the classes H_16_ and H_15_.

**Table 2:** The Distribution of Human, Fly, and Human Homologs of Mouse Essential Proteins Among H_i_ Classes.

H_i_	No. of Human Proteins	No. of Fly Proteins	No. of Mouse Homologs Proteins
**17**	261 (84%)	1119 (91%)	1849 (85%)
**16**	20 (6.4%)	58 (4.7%)	141 (6.4%)
**15**	18 (5.7%)	8 (0.65%)	44 (2%)
**14**	2	7	42
**13**	2	3	24
**12**	6	1	32
**11**	2	3	24
**10**	0	8	12
**9**	0	1	0
**8**	0	2	3
**7**	0	0	2
**6**	1	7	2
**5**	0	3	0
**4**	0	5	0
**3**	0	2	0
**2**	0	1	0
**1**	0	0	0
**0**	0	0	0

Mouse essential genes were recently studied in Georgi et al., 2013 [18]. In this paper, the set of their human orthologous genes was also determined. We examined how these human orthologous of mouse essential genes are distributed with respect to the different conservation classes (see Table 2). The pattern is similar to that of both human and fly essential genes, and in particular, the vast majority of these genes are universal.

### Functional Enrichment: Universal and Non-universal Proteins

Approximately 65% of both human and fly RefSeq proteins are universal. A GO term is enriched among a class of proteins with respect to a “background” set (e.g. the universal proteins with respect to all proteins) if the proportion of proteins in the class having its annotation is noticeably higher than the proportion of this GO term in the background set. With very high probability, a randomly chosen proteins subset will have no significant enrichment of gene ontology (GO) terms. A central question we asked is whether the set of universal proteins, as well as its complement, behave in that respect as random subsets of all proteins, or if they have significant GO terms enrichment. We found out that there are numerous GO terms in all 3 GO categories - biological processes (BP), molecular function (MF), and cellular component (CC), that are significantly enriched in both human and fly universal proteins, and there are some significant enrichments among non-universal proteins as well. Thus, the sets of universal proteins and the sets of non-universal proteins differ substantially from random subsets with respect to GO enrichment results.

**Figure 1:**
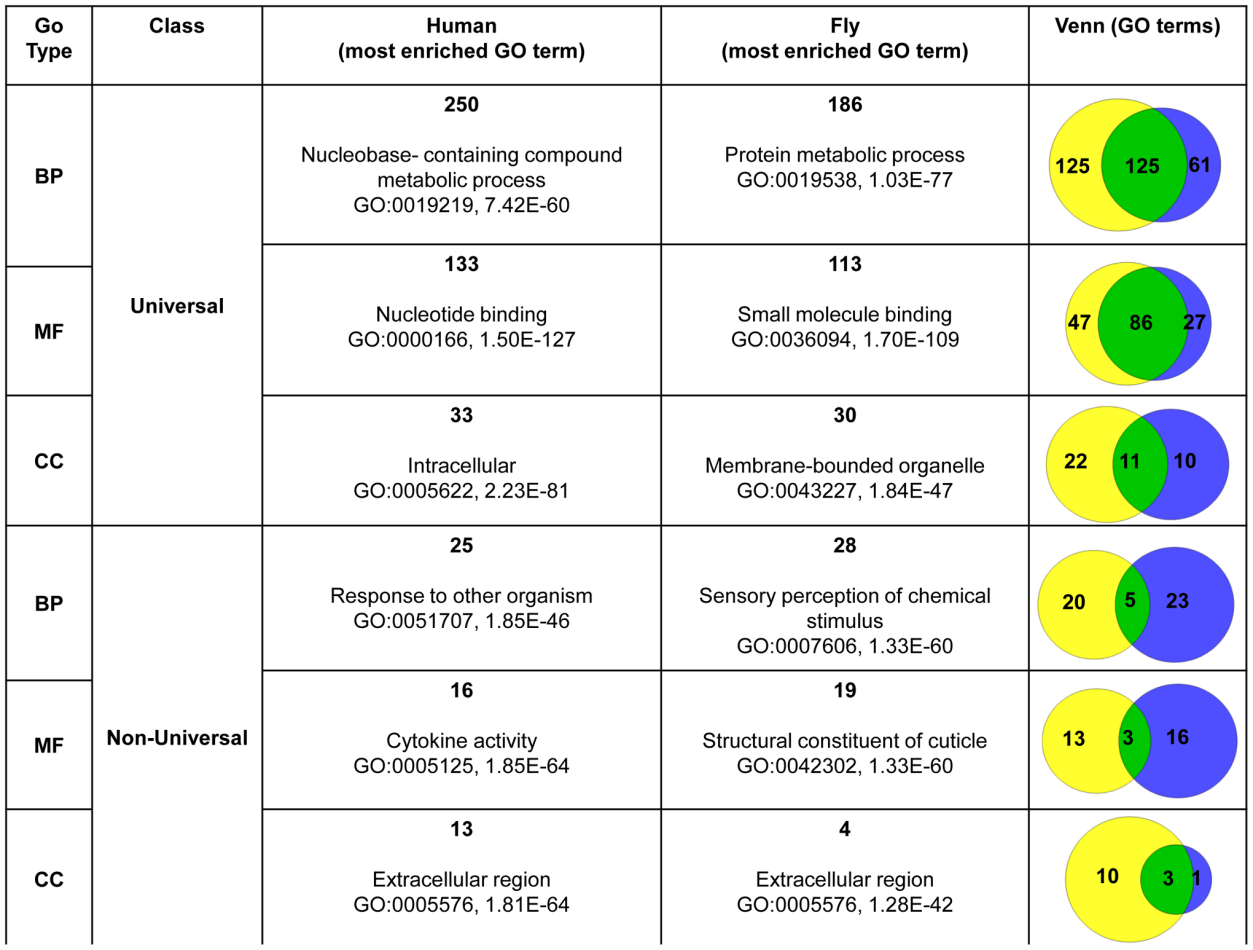
Number of Significantly Enriched GO Terms within Universal and Non-Universal Proteins Classes. The figure describes the number of significantly enriched GO terms (p-value 10^−6^ or smaller) in human and fly universal and non-universal proteins. Each class is divided to the three major GO categories: biological process (BP), molecular function (MF), and cellular component (CC). Within each of the resulting 12 categories, the GO term with most significant p-value is described. The Venn diagrams depict, for each category, the number of terms shared between human and fly, and those unique to these two species. Human GO terms appear in yellow, fly GO terms appear in blue, intersection appears in green.

Figure 1 summarizes GO terms enriched with p-values less than 10^−6^ for human and fly universal and non-universal proteins. In each category, the most significant term and its p-value are also given. The rightmost column shows Venn diagrams intersecting all enriched human and fly GO terms for each category.

The nature of enriched terms and their number is fairly similar for human and fly universal proteins, but differs for non-universal ones. When considering the abundance of enriched GO terms, there is a marked difference between the classes of universal and non-universal proteins. At any level of significance (namely p-values for hypergeometric test), the universal proteins contain substantially more enriched terms than the non-universal ones. As a specific example, there are tens of BP terms that are enriched with p-values smaller than 10^−10^ among human universal proteins, but there are only 5 such terms in human non-universal proteins.

We first examine the GO terms that are enriched among both human and fly universal proteins. There are 125 such terms in the BP category, 86 terms in the MF category, and 11 at the CC category. We concentrate on the “top” of the list – those GO terms enriched with p-value 10^−20^ (or smaller) in both human and fly universal proteins. There are 22 such BP terms (Table 3), 31 MF terms (Table 4), and 2 CC terms (Table 5). For biological processes, the highly enriched terms are related to regulation of transcription, regulation of specific metabolic processes, protein modification process, signal transduction, phosphorus metabolic process (protein phosphorylation), proteolysis, establishment of localization and transport. For molecular functions, the highly enriched terms are related to nucleotide binding, zinc ion binding, ATP binding, transcription factor activity, protein kinase activity, and peptidase activity. Intracellular and plasma membranes are the two most significantly enriched cellular component terms. These functions are generally considered to be central to all life forms.

**Table 3:** Biological Process GO Universal Terms and Their Enrichments (p-values)* ** ***.

GOID	Description	Human H17	Human NU	Fly H17	Fly NU
**GO:0019219**	regulation of nucleobase-containing compound metabolic process	7.42E-60	None	3.41E-28	None
**GO:0051171**	regulation of nitrogen compound metabolic process	1.02E-58	None	1.35E-28	None
**GO:0051252**	regulation of RNA metabolic process	1.40E-52	None	1.14E-26	None
**GO:0006355**	regulation of transcription, DNA-dependent	3.58E-51	None	1.72E-21	None
**GO:2001141**	regulation of RNA biosynthetic process	9.54E-51	None	1.72E-21	None
**GO:0019538**	protein metabolic process	9.91E-50	None	1.03E-77	None
**GO:0035556**	intracellular signal transduction	4.74E-46	None	1.56E-22	None
**GO:0010468**	regulation of gene expression	2.82E-43	None	6.09E-26	None
**GO:0006464**	cellular protein modification process	1.48E-42	None	5.42E-44	None
**GO:0036211**	protein modification process	1.48E-42	None	5.42E-44	None
**GO:0044267**	cellular protein metabolic process	2.09E-38	None	1.59E-37	None
**GO:0043412**	macromolecule modification	8.41E-38	None	3.44E-39	None
**GO:0007165**	signal transduction	2.11E-33	None	8.45E-25	None
**GO:0006796**	phosphate-containing compound metabolic process	7.64E-33	None	1.36E-35	None
**GO:0006793**	phosphorus metabolic process	7.64E-33	None	1.36E-35	None
**GO:0006508**	proteolysis	4.00E-30	None	6.77E-43	None
**GO:0006468**	protein phosphorylation	1.31E-28	None	4.46E-28	None
**GO:0044260**	cellular macromolecule metabolic process	3.22E-28	None	1.47E-39	None
**GO:0051234**	establishment of localization	3.27E-28	None	1.03E-42	None
**GO:0006810**	transport	4.35E-28	None	3.70E-41	None
**GO:0055085**	transmembrane transport	1.71E-27	None	5.05E-28	None
**GO:0016310**	phosphorylation	3.04E-27	None	3.24E-27	None

*Human Universal Proteins (Human H17), Human Non-Universal Proteins (Human NU), Fly Universal Proteins (Fly H17) and Fly Non-Universal Proteins (Fly NU).

**‘None’ notation indicates no significant enrichment was detected (with p-value larger than 10^−2^).

***Only GO terms that are enriched with p-value 10^−20^ or smaller in H17 for both human and fly are presented.

**Table 4:** Molecular Function Universal GO Terms and Their Enrichments (p-values)* ** ***.

GOID	Description	Human H17	Human NU	Fly H17	Fly NU
**GO:0000166**	nucleotide binding	1.50E-127	None	7.33E-106	None
**GO:0036094**	small molecule binding	1.59E-119	None	1.70E-109	None
**GO:0032553**	ribonucleotide binding	6.43E-114	None	1.02E-86	None
**GO:0035639**	purine ribonucleoside triphosphate binding	7.95E-114	None	1.46E-86	None
**GO:0032555**	purine ribonucleotide binding	8.45E-114	None	1.02E-86	None
**GO:0008270**	zinc ion binding	5.63E-111	None	1.57E-58	None
**GO:0017076**	purine nucleotide binding	2.05E-109	None	1.22E-86	None
**GO:0046914**	transition metal ion binding	3.85E-94	None	1.13E-69	None
**GO:0032559**	adenyl ribonucleotide binding	4.41E-86	None	9.50E-65	None
**GO:0005524**	ATP binding	2.44E-85	None	1.34E-64	None
**GO:0030554**	adenyl nucleotide binding	5.34E-83	None	5.78E-64	None
**GO:0003676**	nucleic acid binding	1.62E-80	None	1.32E-52	None
**GO:0003677**	DNA binding	5.68E-72	None	9.24E-21	None
**GO:0017111**	nucleoside-triphosphatase activity	2.43E-61	None	1.68E-39	None
**GO:0016462**	pyrophosphatase activity	3.83E-60	None	1.45E-38	None
**GO:0043565**	sequence-specific DNA binding	1.96E-59	None	1.04E-26	None
**GO:0016818**	hydrolase activity, acting on acid anhydrides, in phosphorus-containing anhydrides	6.24E-58	None	1.70E-36	None
**GO:0016817**	hydrolase activity, acting on acid anhydrides	1.27E-56	None	4.31E-36	None
**GO:0001071**	nucleic acid binding transcription factor activity	1.72E-56	None	1.11E-33	None
**GO:0003700**	sequence-specific DNA binding transcription factor activity	2.22E-56	None	1.11E-33	None
**GO:0004672**	protein kinase activity	9.39E-49	None	1.44E-29	None
**GO:0016301**	kinase activity	5.35E-47	None	3.81E-26	None
**GO:0016773**	phosphotransferase activity, alcohol group as acceptor	2.70E-46	None	2.00E-27	None
**GO:0004674**	protein serine/threonine kinase activity	4.15E-40	None	6.50E-24	None
**GO:0016787**	hydrolase activity	1.13E-32	None	1.51E-51	None
**GO:0005525**	GTP binding	1.00E-27	None	1.27E-23	None
**GO:0019001**	guanyl nucleotide binding	1.78E-27	None	6.10E-24	None
**GO:0032561**	guanyl ribonucleotide binding	1.78E-27	None	1.27E-23	None
**GO:0004175**	endopeptidase activity	2.42E-23	None	1.63E-35	None
**GO:0070011**	peptidase activity, acting on L-amino acid peptides	2.47E-21	None	2.72E-41	None
**GO:0008233**	peptidase activity	4.61E-21	None	2.64E-36	None

*Human Universal Proteins (Human H17), Human Non-Universal Proteins (Human NU), Fly Universal Proteins (Fly H17) and Fly Non-Universal Proteins (Fly NU).

**‘None’ notation indicates no significant enrichment was detected (with p-value larger than 10^−2^).

***Only GO terms that are enriched with p-value 10^−20^ or smaller in H17 for both human and fly are presented.

**Table 5:** Cellular Components Universal GO Terms and Their Enrichments (p-values)* ** ***.

GOID	Description	Human H17	Human NU	Fly H17	Fly NU
GO:0005622	intracellular	2.23E-81	None	4.62E-21	None
GO:0005886	plasma membrane	1.06E-43	None	1.66E-22	None

*Human Universal Proteins (Human H17), Human Non-Universal Proteins (Human NU), Fly Universal Proteins (Fly H17) and Fly Non-Universal Proteins (Fly NU).

**‘None’ notation indicates no significant enrichment was detected (with p-value larger than 10^−2^).

***Only GO terms that are enriched with p-value 10^−20^ or smaller in H17 for both human and fly are presented.

Compared to human and fly universal proteins, there are far fewer GO terms that are enriched with p-value 10^−6^ (or smaller) in non-universal proteins of both human and fly (Figure 1): these are 5 BP terms, 3 MF terms, and 3 CC terms (Table 6). The following terms are enriched among the non-universal proteins of both human and fly: BP jointly enriched terms are response to other organism, response to biotic stimulus, response to bacterium, defense response to bacterium, and defense response. MF jointly enriched terms are hormone activity, neuropeptide hormone activity, and receptor binding, and CC jointly enriched terms are extracellular region, extracellular region part, and extracellular space. The proteins associated with these functions are highly variable and therefore most of them are not universal.

**Table 6:** GO Terms Enriched in Non-Universal Proteins (and their p-values)* ** ***.

BP ID	Description	Human H17	Human NU	Fly H17	Fly NU
**GO:0051707**	response to other organism	None	1.10E-19	None	2.17E-11
**GO:0009607**	response to biotic stimulus	None	6.69E-16	None	3.07E-11
**GO:0009617**	response to bacterium	None	2.57E-15	None	2.68E-09
**GO:0006955**	immune response	None	1.17E-14	None	4.47E-07
**GO:0006952**	defense response	None	1.18E-12	None	2.22E-08
**MF ID**					
**GO:0005179**	hormone activity	None	1.05E-35	None	6.71E-21
**GO:0005102**	receptor binding	None	2.22E-13	None	6.68E-07
**GO:0005184**	neuropeptide hormone activity	None	4.76E-13	None	4.59E-13
**CC ID**					
**GO:0005576**	extracellular region	None	1.81E-64	None	1.28E-42
**GO:0005615**	extracellular space	None	8.51E-21	None	1.64E-12
**GO:0044421**	extracellular region part	None	4.74E-17	None	1.40E-14

*Human Universal Proteins (Human H17), Human Non-Universal Proteins (Human NU), Fly Universal Proteins (Fly H17) and Fly Non-Universal Proteins (Fly NU).

**‘None’ notation indicates no significant enrichment was detected (with p-value larger than 10^−2^).

***Only GO terms that are enriched with a p-value of 10^−6^ or smaller in NU for both human and fly are presented.

There are a number of GO terms enriched with p-value smaller than 10^−6^ in either human or fly non-universal proteins, but are not enriched in the other reference species (see Venn diagrams in Figure 1). For example, terms enriched only in fly non-universal proteins (i.e. unique fly enriched functions) include sensory perception of taste and of smell, chitin metabolic process, and body morphogenesis (BP). The human unique terms include keratinization, calcium-independent cell-cell adhesion, response to type I interferon, and inflammatory response (BP). Complete data for BP, MF and CC is available in Table S1. These functions demonstrate enrichment of specific terms, some of which are known in the literature, while others are novel and hypothetical.

### Distribution of Homologues for Specific GO Terms

**Figure 2:**
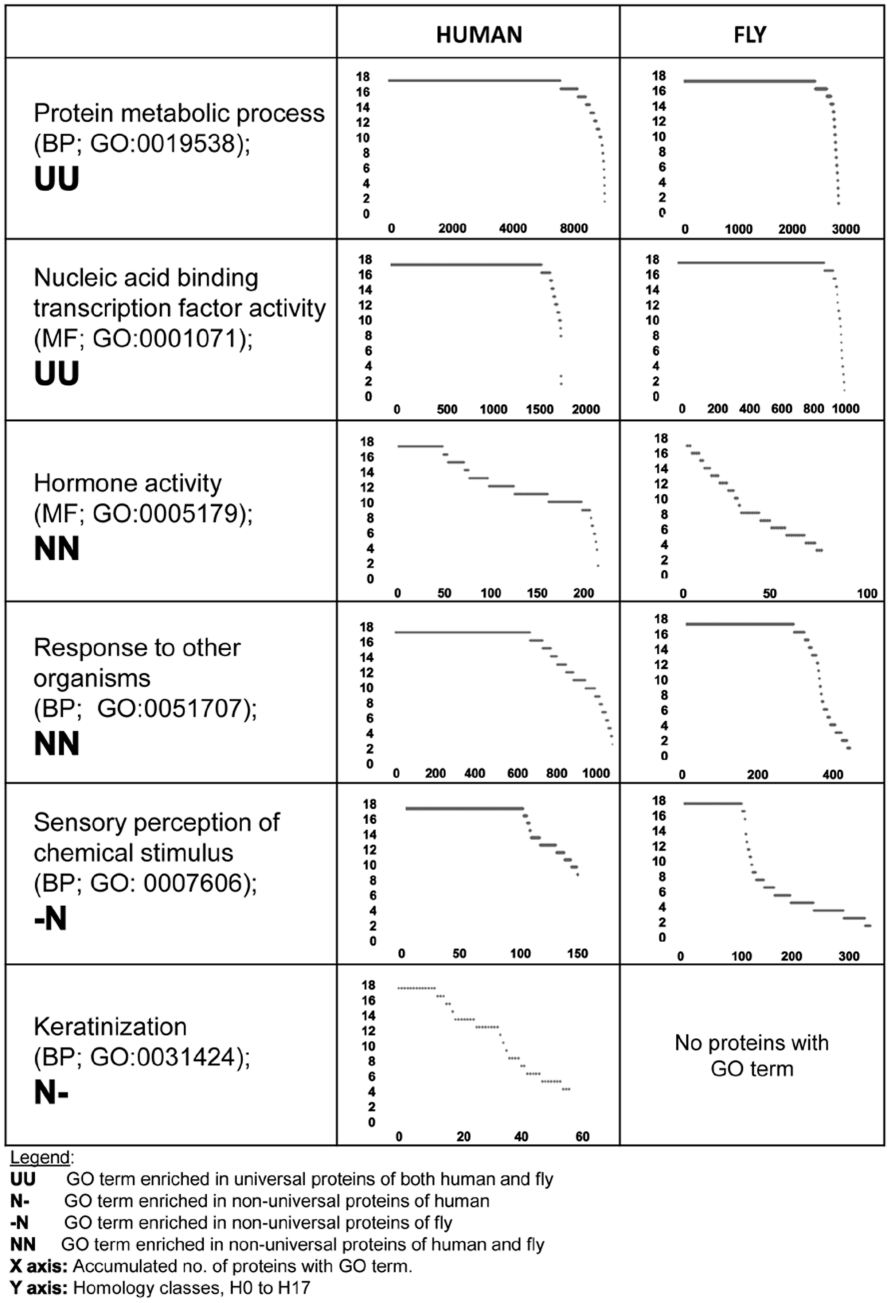
Histograms of Conservation Profiles for 6 Selected GO Terms. Histograms of conservation profiles for 6 selected GO terms are presented. The vertical axis denotes the H_i_ classes, corresponding to the number of species with a homologue for a reference-protein (H_0_ to H_17_). The horizontal axis denotes the accumulated number of reference proteins with the selected GO term, in the corresponding H_i_ class. Each row in the table shows two histograms: one from the human point of view and the other from the fly's. UU are GO terms that are enriched in **U**niversal-protein in both human and fly. NN are GO terms that are enriched in **N**on-universal proteins in both human and fly. N-/-N are GO terms that are enriched in either human's or fly's (respectively) **N**on-universal proteins, but not in both.

We have examined six specific GO terms of interest in more detail, examining how the numbers of homologues for proteins within these terms are distributed (Figure 2). In this figure, all proteins listed under each of these 6 selected GO terms, were ordered by their conservation classes. The first two terms, protein metabolic process (BP), and nucleic acid transcription factor activity (MF) are enriched among both human and fly universal proteins. We denote this by UU. The next two terms, hormone activity (MF) and response to other organisms (BP) are enriched among both human and fly non-universal proteins (NN). Finally, sensory perception of chemical stimulus (BP) is enriched among fly non-universal proteins only (-N), and keratinization (BP) is enriched among human non-universal proteins only (N-). The histograms for the first two terms are quite similar: a high proportion of proteins are universal (17 homologues), and then a very sharp drop with fewer and fewer homologues. The remaining 4 GO terms exhibit a smaller accumulated number of universal proteins, and then a larger proportion of non-universal proteins. Proteins with hormone activity annotation, in particular, exhibit an almost uniform distribution among the various numbers of homologues (H_i_ classes), a very different pattern than the one observed in the first two terms.

### TOL Consistent and TOL Inconsistent Proteins: Functional Enrichment

There are numerous conservation profiles for non-universal proteins. We employed the tree of life (TOL) to distinguish between profiles that are compatible with the accepted species phylogeny from incompatible ones. The TOL project [19] aims to display the exact phylogenetic history of numerous species. There are still some controversies about major and minor branches in the TOL. However, when restricted to our 18 metazoans, which are all well explored model organisms, there is a wide agreement on the TOL.

We define a non-universal protein to be TOL consistent if either all species where the protein has homologues form a monophyletic clade in the TOL, or if all species where the protein does not have homologues form a monophyletic clade in the TOL (see methods). For example, fly proteins that have homologues in all invertebrates but not in any vertebrates are TOL consistent. There are just 5 such proteins. By way of contrast, there are 287 human proteins that have homologues in all vertebrates but not in any invertebrates, and are labeled as TOL consistent. Taking the background set as all non-universal proteins, these 287 human proteins are enriched with various top level BP functions, mainly response to stimulus and signaling (data not shown). MF enrichment of these proteins includes receptor binding (G-protein, cytokine and chemokine). It was recently reported [20] that cytokine-like (e.g. L-2, IL-8) and chemokine molecules were found in invertebrates, yet with extreme sequence variability, despite structure and function conservation. There are 862 human proteins with homologues in all species, except the worm. They are non-universal, TOL consistent, and are enriched (with respect to the same background) for transcription (BP) and sulfotransferase activity (MF) (data not shown). Likewise, fly proteins having homologues in all species except the worm are non-universal and TOL consistent. There are 491 such proteins, and they are enriched (with respect to the same background) for GO terms that include lipid metabolic process (BP) and hydrolase and lipase activities (MF) (data not shown).

Overall, there are 2,221 non-universal TOL consistent fly proteins (out of 7,865 non-universal fly proteins), and 3,869 TOL consistent non-universal human proteins (out of 11,976 non-universal human proteins). The default BLAST threshold used for determining presence of homologues is very low, resulting in approx. 65% of all proteins being universal. TOL consistency of non-universal proteins, on the other hand, is a strict criterion, resulting in just 30% of all non-universal proteins being TOL consistent. This indicates that TOL consistency “corrects” for spurious homologies stemming from the lenient BLAST homology threshold.

We examined functional enrichment in the non-universal TOL consistent and the TOL inconsistent proteins of both human and fly. In all cases, the background was the set of all non-universal RefSeq proteins of the corresponding species. There are many more enriched GO terms among TOL consistent proteins than among TOL inconsistent ones, their p-values are more significant, and the numbers of proteins per term are generally larger.

TOL consistent non-universal fly proteins exhibit strong GO enrichment for a large number of terms, including metabolic process, cellular process, and transport (BP) catalytic activity, hydrolase activity (MF) intracellular part, organelle part (CC). TOL consistent non-universal human proteins are strongly enriched for metabolic process, cellular process, and gene expression (BP), catalytic activity and binding (MF), and intracellular part and cytoplasmic part (CC) (Table S2, where non-human or fly proteins served as the background, correspondingly).

TOL inconsistent non-universal human proteins exhibit very few substantially enriched GO terms (Table S3, where non-universal human or fly proteins served as the background, correspondingly). The few enriched human terms include regulation of immune response (MP) and extracellular region (CC). Fly enriched terms include sensory perception (MP), structural constituent of cuticle (BF), and extracellular region (CC).

### Quantum Clustering of Non-universal, TOL Consistent Proteins

We clustered the conservation profiles of TOL consistent non-universal proteins, using the Quantum Clustering algorithm (see Methods). The input to the clustering algorithm is the set of conservation profiles (each is a binary vector with 17 entries), and the multiplicity of each pattern. The conservation profiles of all (universal and non-universal) proteins appear in Table S3 (for both human and fly). Using standard settings, the Quantum Clustering algorithm produced 9 clusters for both human and fly TOL consistent non-universal proteins. In addition to the above-mentioned data, the resulting mapping from profiles to clusters is described in Table S4 as well.

**Figure 3:**
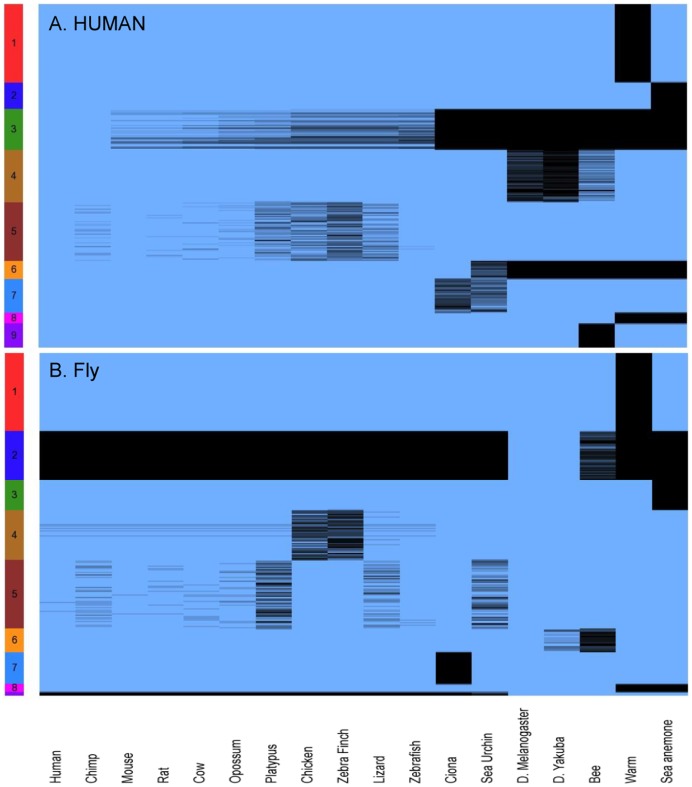
Conservation Profiles for Clusters of Non-Universal TOL Consistent Proteins (human, A; fly, B). Conservation profiles for clusters of non-universal TOL consistent proteins (produced using QC) for human (9 clusters, A); and Fly (9 clusters, B). Each row contains the conservation profile for a reference protein, across all 17 other species (columns). Proteins are grouped in clusters (leftmost rainbow colored column), as the outcome of the Quantum Clustering (QC). Blue indicates presence of a homologue in a certain species, black indicates its absence.

Figure 3 describes graphically the mapping from profiles to clusters (A. Human, B. Fly). The rows correspond to the conservation profiles of proteins in the different clusters, and the columns correspond to the species. Blue indicates the presence of a homologous protein in the corresponding species, while black indicates its absence. Human cluster 1 has 862 proteins with a single conservation profile: All these human proteins have homologues in all species, except the worm. Human cluster 5, on the other hand, has 648 proteins with 11 conservation profiles, characterized primarily by missing homologues in zebra finch, chicken, lizard, and platypus. Fly cluster 1 has 491 proteins, again with a single conservation profile: All these fly proteins have homologues in all species, except the worm. Fly cluster 2 has 308 proteins, with 2 conservation profiles. All these proteins have homologues in Drosophila yakuba, and some have homologues in bee. Fly cluster 9, the smallest cluster, has only 23 proteins, with 2 different profiles. The fly proteins in this cluster have homologues in Drosophila yakuba, bee, sea anemone, and worm.

For each cluster, we computed the “center of mass” of all conservation profiles in it, taking multiplicity into account. We have also computed the most abundant profile, termed MAP (Table S4, under the stats tabs). For most clusters, the entries in most centers of mass are “close to binary”: above 0.85, or below 0.15. This indicates a measure of coherence of profiles in clusters. Yet some clusters, like human cluster 7 or fly cluster 6, have entries that are not close to binary. The worm, Caenorhabditis briggsae, and the sea anemone, Nematostella vectensis, seems to play a special role in all clusters. In every human cluster and every fly cluster, all worm entries in the clusters are uniform (all are 1 or all are 0), and so are all sea anemone entries. We note that there are clusters where the proteins of these two species do not agree. For example, both fly and human protein cluster 1 contain proteins that have homologues in all species, including sea anemone, but have no worm homologues. Table S5 describes this information concisely with respect to each human and fly cluster, correspondingly. The tables describe the number of proteins, number of different profiles, most abundant profiles and their multiplicity, and centers of mass per cluster. As indicated above, both human and fly have clusters with a single conservation profile. For example the largest cluster in both fly and human (cluster 1) has a single conservation profile, corresponding to proteins whose homologues are present in all 16 species and are missing (no homologues) in the worm. We remark that from an evolutionary point of view, sea anemone (a cnidarian) is the outgroup among the 18 species. Removing sea anemone, the worm (a nematoda) is the outgroup of the remaining 17 species.

### Functional Enrichment in Clusters of TOL Consistent Proteins

We identified enriched GO terms among the set of proteins in each cluster, using the GOrilla tool, with the TOL consistent non universal proteins serving as the background set. Most of the human and fly clusters terms have GO enrichment with p-value smaller than 10^−5^. Enriched terms for each cluster and additional information are depicted in Table S5.

For example, fly clusters 2 and 9 (BP and MF) present a hierarchy of enriched sensory perception of chemical stimulus terms. This is part of neurological processes that are responsible for the development of smell and taste, which are so important in insects' life-style. In another example, lipid metabolism functions are enriched in fly clusters 1 and 6 (BP), and 1 and 3 (MF). In a recent paper [21], the functions of lipid metabolism were studied beyond the traditional functions of energy storage, parts of membranes and precursors for vitamins and hormones. The drosophila serves as a model for studying human biological questions. In their paper, Liu and Xuang use the drosophila model to study lipid metabolism disorders. The fly clusters above, with enriched terms related to lipid metabolism, may provide additional candidates of proteins for such a study.

Another example are GO terms related to the human eye lens, enriched in human cluster 6 (BP, MF and CC, Table S5). The importance of gap junctions, mediated by connexins in human lens has been widely studied [22], [23]. Despite their functional importance and wide distribution in human (20 proteins in the family), the connexin family is not conserved in evolution, and no sequence homologues are found in invertebrates. Instead, the innexin protein family, having the same 4-transmembrane topology but no sequence similarity to the connexins exists (in lower numbers).

Finally, we observe that human cluster 9 contains proteins that have homologues in all species except the bee. This cluster is enriched for sialyltransferase activity (MF). Sialytransferases have long been implicated as being involved in metastasizing tumors in the rat and in immune response [24]. We conjecture that lack of homologues to sialyltransferase activity proteins in the honey bee is related to bee diseases.

## Discussion

Conservation profiles are binary in nature, being based on “presence or absence” patterns of homologous proteins. They were introduced and used in the literature mostly for inferring evolutionary histories [8]–[10]. Conservation profiles were also used for inferring and predicting organism-to-phenotype associations [11]. Martin et al. [14] were the first to use these profiles in a large scale study of bacterial protein families, using the E. coli point of view. Lopez-Bigas et al. [4] studied conservation of 16 genomes, ranging from mammals to fungi, from the human point of view. They were mainly using a quantitative conservation score measure, called CS, and applied both to orthologues and to homologues of all human proteins. Many studies investigate individual protein family or families across many species (e.g. DeVries et al. [25] for the Chemokine/Chemokine Receptor System). Lopez-Bigas et al. [4] study all protein families, assign quantitative score to each, and compare the conservation across the different species. In that respect, Lopez-Bigas et al. [4] study can be described as “bottom up”: going from each protein family individually to the global conservation view.

By way of contrast, our methodology can be described as “top down”. We hierarchically partition the protein space at three levels: (1) universal vs. non universal; (2) TOL consistent vs. TOL inconsistent; and (3) clusters of TOL consistent proteins. Then, for each sub-partition, we identify families with significant GO enrichment. Since the default BLAST homology threshold is very low, a homologous protein need not have the same functionality. It is thus not clear a-priori that the proteins in any of these sub-partitions will have any meaningful enrichment, let alone the abundance of significant enrichments we find. We note that in the context of predicting enzyme functional classes, the use of top-down approach, as opposed to the bottom up investigation of specific families, was advocated by Shen and Chou [26].

A second methodological innovation of our work is the consideration of more than one reference species. Most studies of protein conservation across various sets of organisms take a single species “point of view” (a reference-species). For example, E. coli for sets of bacteria, or human for sets of metazoa. We employ the traditional human point of view, and add a second one, that of D. melanogaster. This approach reveals both similarities and differences between enriched protein families of the two reference species, and their significance. Using both the human's and fly's viewpoints enables us to shed light on conservation characteristics that would not be observable based on a single viewpoint alone.

GO terms that are enriched among the universal proteins of both reference species represent the more robustly conserved and thus, functionally important protein families. These include core processes, such as protein metabolic processes and transport, as well as regulatory processes such as transcription and regulation of gene expression. Our findings for these “doubly enriched” terms bear similarities to the conserved families of Lopez-Bigas et al. [4], yet there are also noticeable differences. For example, protein metabolic process and transport (BP) are significantly enriched in universal human and fly proteins, a finding that is in agreement with [4], but nucleic acid binding and DNA binding (MF) are also significantly enriched in our universal human and fly proteins (see Figure 2, 2^nd^ row), while labeled as divergent in fly orthologues according to [4].

The third methodological innovation is the study of enrichment in the context of TOL consistent (non-universal) proteins. This enables to automatically identify protein families enriched among specific clades in the tree of life. For example, terms enriched among human proteins with homologues in vertebrates but not in invertebrates, or terms enriched among fly proteins having no worm homologues. Human cluster 6, which includes human proteins with homologues in chordate, only exhibits enrichment of visual perception and structural constituent of eye lens; indeed we found references in the literature to the corresponding conservation pattern of the connexin family [22], [23]. Other clusters exhibit strong enrichment, which is not supported by known findings, yet raises putative conjectures. For example, human cluster 2 is enriched for retinal binding, retinoid binding, and carbohydrate binding. These human proteins have homologues in every other species, except sea anemone. Sea anemone, a cnidarian, is an outgroup to the set of other 17 metazoan species, which are all bilaterian metazoans. So this finding supports the hypothesis that many proteins with these functionalities were added in the bilaterian ancestor, after the sea anemone speciation.

Fang et al. [27] describe a daily-updated sequenced/species Tree Of Life (sTOL). They chose individual GO terms, identified all the domains (at the superfamily level) annotated with them, and then examined their presence and abundance in subtrees of the sTOL. This enabled them to analyze the evolutionary dynamics of such GO terms. The approach of Fang et al. [27], like the approach of Lopez-Bigas et al. [4], can also be described as “bottom up”. We use the standard TOL, restricted to our 18 metazoan species, as a reference tree. The conservation profile alone serves to hierarchically partition the human and the fly proteins to subfamilies, and GO enrichment analysis is then globally applied to these partitions, in a “top down” fashion.

The sTOL approach and ours represent very different methodologies. Yet, some of the GO enrichment results are similar, while others are incomparable. For highly conserved sets of proteins, the most significantly enriched terms are related. For example, the most significantly enriched biological process terms in the “present in Eukaryota” category of [27] are (1) cellular metabolic process, (2) biosynthetic process, and (3) primary metabolic process. The most significantly enriched biological process term in our “human and fly universal” category is (4) protein metabolic process. Interestingly, in the GO terms hierarchy, metabolic process is an ancestor of (1), (2), and (3), and (3) is an ancestor of (4), so these terms are closely related [28].

On the other hand, central GO terms such as transport (biological process), ribonucleotide binding and kinase activity (molecular function) are highly significantly enriched among our “human and fly universal” (see Table 6), but are not included in the Fang et al. [27] list of enriched terms. Furthermore, our “human and fly non-universal” category (see Table 6) includes the significantly enriched terms response to biotic stimulus (biological process) and hormone activity (molecular function), which are also not part in the Fang et al. [27] list of enriched terms. In fact, this and other categories of ours do not have parallel ones in [27].

Our GO analysis results have led to some novel insights. A rather unexpected class includes GO terms enriched among non-universal proteins of both reference species. This class includes immune response, defense response, and related terms (e.g. response to biotic stimulus, BP), and hormone activity and receptor binding (MF). These GO terms represent processes or functions whose roles are preserved, yet individual proteins in each species vary substantially, thus most of them are not universal. Proteins involved in hormone activity are highly variable and possess a wide distribution of conservation profiles, from both points of view (Figure 2, 3^rd^ row). Hormones and their receptors are known as specific regulators. These observations are consistent with [4] that reported that proteins involved in defense response, immune response, as well as in regulatory processes, are highly divergent.

Another class of interest includes GO terms enriched among non-universal proteins in one reference species, but not the second. These represent processes or functions that were specifically developed in one reference species but not in the other. For example, keratinization, response to type I interferon, cytokine activity, and chemokine activity are enriched in human non-universal proteins. Sensory perception, chitin metabolic process, structural constituent of cuticle, and odorant binding are enriched in fly non-universal proteins. This characterization of these terms is supported by various biological findings: Keratins are only found in genomes of chordates, so for example, human have 2 gene clusters harboring 25 keratin genes each [29], whereas Ciona has only one pair of keratin genes, and D. melanogaster has none. The interferon system plays an important role in resistance to viral and bacterial infections. Interferon genes are highly variable, with poor homology between mammalian and non-mammalian vertebrate genes, and are found only in vertebrates [30], [31]. Chitin has a unique role in insects, both in building the exoskeleton, and as a basis for morphogenesis [32]. Sensory perception and odorant binding are basic, crucial functions in the fly's life, involved in locating food and identifying danger. These proteins are also highly divergent [33].

Finally, there are a handful of GO terms, which are enriched among the universal proteins of one species and among the non-universal proteins of the other. Among these are multicellular organismal process (BP) and olfactory receptor (OR) activity (MF) (both enriched in human universal proteins and in fly non-universal proteins). Examining the latter term, there are 59 fly OR proteins, none of which is universal. Indeed, it is known that the fly has a small olfactory receptor protein repertoire [34]. Human has 372 olfactory receptor proteins, which are universal. Other than very few exceptions with marginal homology scores, these homologues are not fly olfactory receptor proteins. Their homologous fly proteins belong to various transmembrane protein families (e.g. serotonin receptor, dopamine receptor, rhodopsins, etc.). Moreover, it was recently published that the fly's olfactory system is based not only on OR but also on other protein families: ionotropic glutamate-like receptors (IRs) and gustatory receptors (GRs), which makes them more sensitive and compensate for the small OR repertoire [35].

The fact that there are about twice as many universal proteins than non-universal ones does not explain the much larger abundance of enriched GO terms among universal proteins than non-universal ones (both for human and for fly). A plausible explanation is the fact that there is a huge variety of conservation patterns for non-universal proteins, while only one pattern for universal proteins. The non-universal proteins are a mixture of many diverse profile populations, and only a few terms (such as those related to defense or immune response) are strong enough to be significant even under such “dilution”. In order to shed light on the gene ontology terms in the non-universal protein population, we focused on TOL consistent proteins, and partitioned their conservation profiles, using quantum clustering, to a small number of non-hierarchical clusters. These clusters are naturally less divergent, and indeed almost all of them exhibit a richer repertoire of enriched GO terms. Furthermore, the clusters enable us to “zoom in” on specific “life style” properties that are related to the few clades, which dominate the clusters. These contain both properties whose role in the clade is well understood, as well as hypothetical ones.

## Methods

### Data Collection and Conservation Profiles

**Figure 4:**
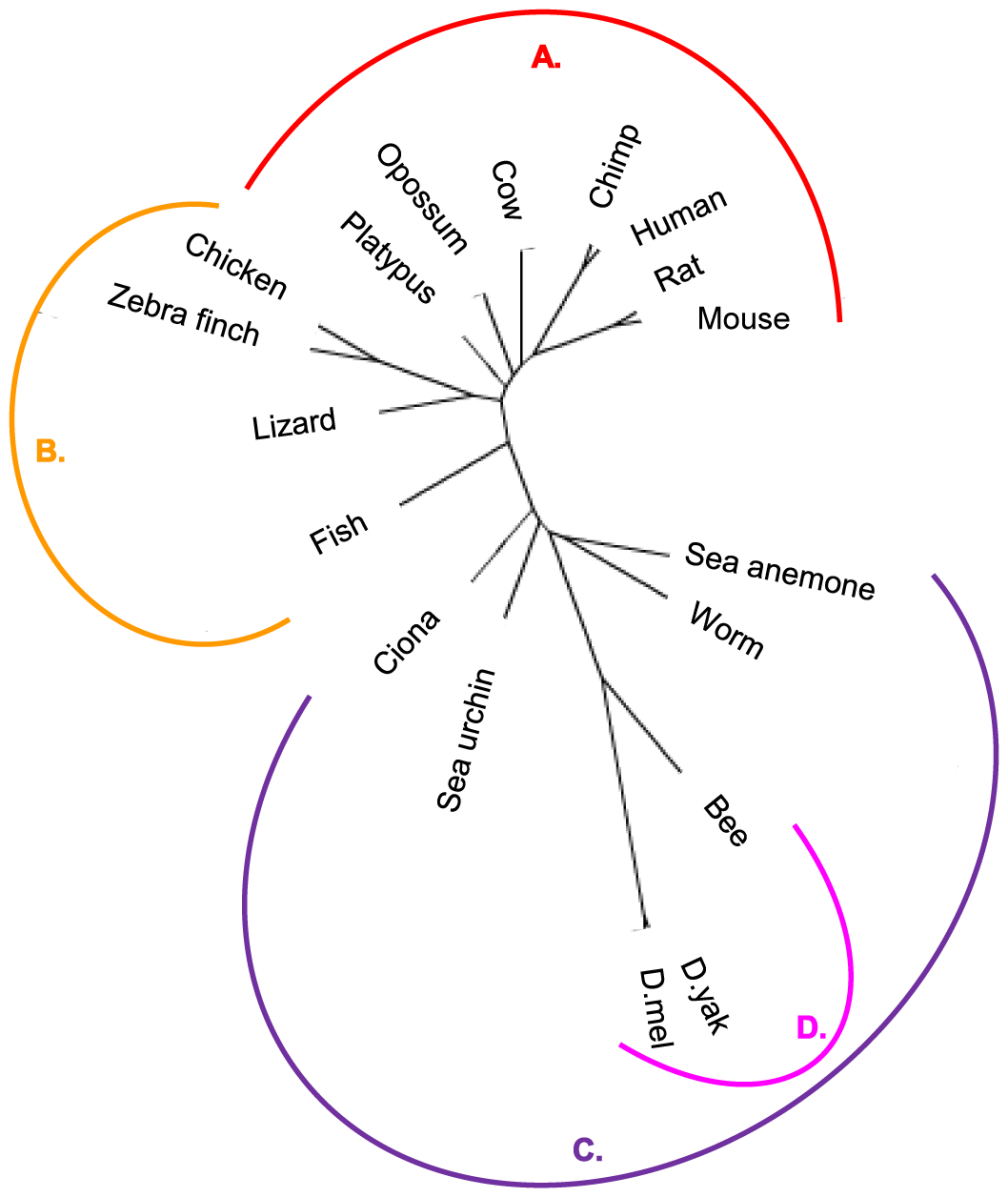
Phylogeny Tree Depiction of All 18 Metazoan Used in This Study. Phylogeny tree depiction of all 18 metazoan used in this study. Divergence points are scaled to time of occurrence. Species are partitioned to four main groups: (A) mammals; (B) non-mammalian metazoans; (C) invertebrates; and (D) insects (Arthropoda). Species Taxonomy ID was obtained from NCBI database, and was inserted to the Interactive Tree Of Life (iTOL) [19]. The resulting tree (in Newick format) was visualized using FigTree: (http://tree.bio.ed.ac.uk/software/figtree/). We note that the visualized tree is unrooted. When rooting the tree, sea anemone (a cnidarian) is the outgroup. All other species are bilaterians.

We studied protein conservation among 18 metazoan species with high quality annotated genomes and proteomes. These taxa (Figure 4) include (A) 7 mammals: human (*Homo sapiens*), chimpanzee (*Pan troglodytes*), house mouse (*Mus musculus*) brown rat (*Rattus norvegicus*), cow (*Bos taurus*), opossum (*Monodelphis domestica*), and platypus (*Ornithorhynchus anatinus*); (B) 4 non-mammalian vertebrates: chicken (*Gallus gallus*), zebra finch (*Taeniopygia guttata*), lizard (*Anolis carolinensis*), and zebrafish (*Danio rerio*); and (C) 7 invertebrates: ciona (*Ciona intestinalis*), sea urchin (*Strongylocentrotus purpuratus*), worm (*Caenorhabditis briggsae*), sea anemone (*Nematostella vectensis*) and 3 isects: bee (*Apis mellifera*), and 2 fruit flies (*Drosophila melanogaster* and *Drosophila yakuba*).

Species Taxonomy ID was obtained from NCBI database, and inserted to the Interactive Tree Of Life (iTOL) [19]. The resulting tree (in Newick format) is visualized, using FigTree (http://tree.bio.ed.ac.uk/software/figtree/) (Figure 4).

From this metazoan set, two well studied reference species were chosen: H. sapiens and D. melanogaster, each accompanied by an evolutionary close species: P. troglodytes (divergence from human, 6 million years ago) and D. yakuba (divergence from D. melanogaster, 10 million years ago). The complete list of RefSeq proteins for these two species (34,817 human sequences, 22,309 fly sequences) were downloaded from NCBI protein database (ftp://ftp.ncbi.nlm.nih.gov/refseq/release/complete/, release 47). Protein BLAST [36] (BLASTP, NCBI version 2.2.25) was locally used to query each of these human and fly Refseq proteins. BLAST queries were run using default scoring parameters. In order to detect even low-scoring homologies for distant species, we set BLAST to retrieve and list the maximum number of alignments per protein. This allowed us to populate every member of a protein binary vector, leaving zeroed members only where no homology was found. In addition, each BLAST query was confined to pairwise align the query protein (i.e. a protein of one of our reference species) against a pre defined list of proteins, containing only proteins of the other 17 metazoan species from the aforementioned set.

Initial hit lists, output by BLAST, were parsed so each reference protein (human's or fly's) was matched with a set of best scoring (achieving highest bit score) homologue proteins, one per species. In cases where no significant homology was detected by BLAST (within default thresholds limits) we assigned that reference protein with a zero score for the queried species.

Having lists of best matching proteins for every reference protein, we assigned each reference protein with a binary vector, of dimension 17, containing ‘1’ for every species which has a homologue (regardless of homology score) and ‘0’ for species where homologues were not found. This vector is also known as the conservation profile, or conservation pattern of a protein with respect to the other 17 species.

Homologous proteins detected for each reference protein were counted, thus assigning each reference protein within a so called conservation class, denoted by H_i_. Thus, proteins spread over a scale ranged between 17 and 0, where H_i_ = 17 represents the existence of a homologue in every species of the 17 within the set (universal proteins), H_i_<17 represents the existence of homologues in a partial set of the 17 species (non-universal proteins) and specifically, H_i_ = 0 represents the special case where no species has a significant homology to the reference protein (orphan proteins).

Using NCBI protein data repository (dated to: Feb. 17^th^, 2012. available at: ftp://ftp.ncbi.nih.gov/gene/DATA/) we extracted essential proteins for both human and fly from the supplement table of MacArthur et al. [17]. Gene symbols in the supplement table were cross-referenced with their RefSeq protein accessions and gene ID. This allowed us to associate essential proteins with H_i_ classes.

The 2,472 gene symbols of Georgi et al. [18] were converted to their corresponding RefSeq IDs, using Alibés et al. [37]. Entries having more than a single variant were truncated to a single one. This process resulted in 2,175 human RefSeq IDs, which were subsequently analyzed to determine their Hi classes.

### Functional Enrichment and Venn Diagrams

For each pair of corresponding target and background sets of proteins (e.g. universal fly proteins vs. all fly proteins, TOL consistent non-universal human proteins vs. all non-universal proteins, cluster 5 fly proteins vs. TOL consistent non-universal fly proteins, etc.), functional enrichment results were obtained using the GOrilla tool [15]. The GO enrichment analysis was carried out in the “two lists mode”, using the target and background lists. We refer to GO terms as significantly enriched if they achieved p-value scores of at most 10^-5^ (yet for completeness, entries with p-values up 10^−4^ are given in the table). Proteins for specific GO enriched terms were extracted from the Gene Ontology Database [38].

Venn diagrams were produced using Venn diagram plotter tool (v1.4.3740) (available at http://omics.pnl.gov/software/VennDiagramPlotter.php).

### TOL Consistent Conservation Profiles

A conservation profile is TOL consistent if either the species having homologous proteins form a monophyletic clade in the TOL, or the species with no homologous proteins form a monophyletic clade in the TOL. In particular, a protein with no homologue in a single species is always TOL consistent. This is equivalent to the existence of a single ancestral node in the TOL such that either the species with homologues are all the descendants of this node, or the species with homologues are all the descendants of this node. The number of non trivial monophyletic clades in our 18 species tree is just 15. Our program simply goes over all non trivial and trivial clades in this tree, one by one, and checks if a given conservation profile exactly fits any one of them.

We remark that the notion of TOL consistency is similar yet different (stricter) from the Dollo's principle [39]. A protein that has homologues only in a single non trivial clade in the TOL, but not in all species in it, will satisfy the Dollo criteria, yet will not be TOL consistent.

### Quantum Clustering

The clustering method employed is Quantum Clustering (QC) [16]. Its underlying principle is that the density of data points within their feature space is represented by a potential function, 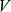. The latter is uniquely derived from a scale-space representation of the data, where a Gaussian with width 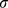 is associated with each one of the data points.

Whereas in the scale-space approach one searches for maxima in the sum of all Gaussians to determine clusters of the data, the QC approach looks for minima of 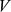. QC employs a gradient-descent procedure to allow the data-points to “move” under an imaginary classical force that is given by 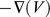, following gradient-descent dynamics. This way the association of data points with clusters is obtained.

QC can be applied to continuous as well as to discrete data. A recommended preprocessing stage involves applying Singular Value Decomposition (SVD), reducing the dimension of the data and representing them as points on a unit-sphere in the chosen dimension. Thus the method involves two parameters: the value of the reduced dimension and the Gaussian width,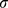. A QC Matlab code is available at http://horn.tau.ac.il/compact.html.

Using SVD for preprocessing we have reduced the data to 5 dimensions. These principal components account for 80% of the variance in the data. The QC parameter was chosen as 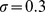 for both human and fly, leading to 9 clusters in both human and fly. We have tested the sensitivity of QC by adding Gaussian noise, 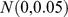, to the binary matrix and verified that the clusters have barely changed. Identical binary vectors in the data are counted independently.

Clusters were visualized using Partek Genomics Suite version 6.5 (Copyright 2010; Partek Inc., MO, USA) from human and fly points of view (Figure 3, A and B, correspondingly).

### Data Availability

In addition to the tables and files available in the paper and in the supporting information, the two larger xlsx files, containing the complete proteomes of human (1A_Human) and fly)1B_D_Melanogaster), with binary conservation profiles and TOL consistency information for each protein, are publicly available at http://www.cs.tau.ac.il/~bchor/TOL_Plos1/ (the folder contains two xlsx files and one readme file).

## Supporting Information

Table S1 **This “four way table” contains GO terms and their enrichments (p-value) for four families of proteins.** (1) human universal proteins (labeled “Human H17”), (2) human non-universal proteins (labeled “Human NH”), (3) fly universal proteins (labeled “Fly H17”) and fly non-universal proteins (labeled “Fly NH”). ‘None’ notation indicates no significant enrichment was detected (with p-value larger than 10^−2^). In these tables, we present only GO terms that are enriched with a p-value of 10^−4^ or smaller in either H17 or NH for at least one species among human and fly. Data is presented in three different tables, as per the three main GO categories: (A) Biological Process; (B) Molecular Function; and (C) Cellular Components.
(XLSX)

Table S2 **Enriched GO terms for TOL consistent non-universal human and fly proteins (with all non-universal proteins of the corresponding species serving as the background set).**
(XLSX)

Table S3 **Enriched GO terms for TOL inconsistent non-universal human and fly proteins (with all non-universal proteins of the corresponding species serving as the background set).**
(XLSX)

Table S4 **This table contains complete data of quantum clustering output for the non-universal TOL consistent proteins of human and fly.** It contains 4 tabs, 2 for human and 2 for fly: the “data” tabs contain quantum clustering assignment for each protein, and the “cluster stats” tabs contain statistics regarding the clusters: most abundant profile (MAP) and center of mass. Each row specifies the total number of proteins inside a QC cluster (2^nd^ column from left), the number of different conservation patterns clustered together (3^rd^ column from left), the most abundant conservation pattern (MAP) in the cluster (4^th^ column), the total number of proteins having the most abundant pattern (5^th^ column), and the calculated center of mass (CM) of all conservation profiles in this cluster (6^th^ column). The remaining columns show the center of mass of all proteins' profiles in this cluster, with respect to each of the other 17 species. The entries are in the range 0 to 1, where 1 indicates that all proteins in this cluster have homologues in the corresponding species, while 0 indicates that none of the proteins in this cluster have homologues in the corresponding species.
(XLSX)

Table S5 **Gene ontology analysis was performed (using GOrilla) on the clusters of TOL consistent non-universal proteins (produced using quantum clustering), which are depicted in** **Figure 3** **.** The results are organized using 3 tabs for each main GO category, for both human and fly. Significantly Enriched GO terms are those with p-value<10^−5^. The background is the set of non-universal TOL consistent proteins of the corresponding species.
(XLSX)
